# A Micro-CT and Synchrotron Imaging Study of the Human Endolymphatic Duct with Special Reference to Endolymph Outflow and Meniere’s Disease

**DOI:** 10.1038/s41598-020-65110-0

**Published:** 2020-05-19

**Authors:** Charlotta Kämpfe Nordström, Hao Li, Hanif M. Ladak, Sumit Agrawal, Helge Rask-Andersen

**Affiliations:** 1 Department of Surgical Sciences, Head and Neck Surgery, section of Otolaryngology, Uppsala University Hospital, Departments of Otolaryngology, Uppsala University Hospital, SE-751 85, Uppsala, Sweden; 2 Department of Otolaryngology-Head and Neck Surgery, Department of Medical Biophysics, and Department of Electrical and Computer Engineering, Western University, London, ON Canada; 3 Department of Otolaryngology-Head and Neck Surgery, and Department of Electrical and Computer Engineering, Western University, London, ON Canada

**Keywords:** Physiology, Medical research, Sensory systems

## Abstract

Meniere’s disease remains enigmatic, and has no treatment with sufficient evidence. The characteristic histopathological finding is endolymphatic hydrops, suggesting either an overproduction or decreased reabsorption of endolymph in the human inner ear. This study presents the first analysis of the vascular plexus around the human endolymphatic duct using micro computed tomography and coherent synchrotron radiation with phase contrast imaging. Using a software program, data were processed by volume-rendering with scalar opacity mapping to create transparent three-dimensional reconstructions. A rich vascular plexus was discovered around the endolymphatic duct that drained into collecting channels, linked to the vestibular venous outflow system. This network is believed to make up the principal route for endolymph outflow, and its associated malfunction may result in endolymphatic hydrops and Meniere’s disease.

## Introduction

The etiology of Meniere’s disease (MD) remains unknown. This inner ear malady is characterised by a symptom triad of episodic vertigo, tinnitus and fluctuant hearing loss. Its unpredictable nature with capricious loss of balance is particularly disabling, influencing the emotional state, mental health and life satisfaction^1^. Environmental factors, ethnicity, gender and age appear to be influencing factors. Its prevalence varies in different investigations from 190 per 100 000 in the United States^2^ to 73–91 per 100 000 for patients 65 years and older^3^. Associations with autonomic nerve and immune system dysfunctions are recognised^4^.

The cause of MD has long been proposed to be an impaired reabsorption of endolymph^5^. This unique fluid bathes the apical poles of the sensory cells, and its proper regulation is essential for inner ear function. The production of endolymph is alleged to occur near the cochlear and vestibular end organs and to “flow” along the membranous duct system to the endolymphatic sac (ES) for reabsorption. The endolymphatic duct (ED) is connected to the ES, which is located in a dura duplicate on the posterior slope of the petrous pyramid near the cerebellum. An overproduction or abridged drain of endolymph in the ES is thought to lead to an accretion or endolymphatic hydrops, which is typical of MD and may explain the symptoms.

The morphology of the ED and ES vary strikingly among different species and in human individuals. The ES is thought to regulate endolymph homeostasis by fluid outflow, cell phagocytosis and pressure monitoring^6–15^. The ES may also play a role in the immune defence of the inner ear^16–21^ which could be etiologically linked to MD^22,23^.

The human ED runs in the vestibular aqueduct (VA), which is about 2.3 mm long (range 1.3–3.4 mm)^24^. It transitions into the ES, which can show large morphological differences. The VA runs parallel to a bony channel, termed the accessory canal (AC) by George Siebenmann in 1874^25^. This canal contains the vein of the vestibular aqueduct (VVA) that drains blood from the vestibular organ. The bony canal was named the para-vestibular canal by Ogura and Clemis in 1971^26^.

In the guinea pig, the ED contains a lympho-venous network located in the sub-epithelial matrix^27^. This network may be physically attached to the epithelium and may drain endolymph. Tracer studies (ionic lanthanum) and freeze fracture transmission electron microscopy show that the ED has shallow epithelial tight junctions that allow para-cellular flux of ions and fluid between cells^28^. Low pressure veins also run in parallel and drain into the cranial sinuses, which are subsequently well-suited for fluid outflow.

Linthicum *et al*.^29^ demonstrated that the human ED is surrounded by a peri-endolymphatic channel system that is assumed to be involved in endolymph resorption. In the present study, we performed a micro-CT and synchrotron phase contrast imaging (SR-PCI) study with three dimensional (3D) reconstructions to establish the organisation of the channel system and its relation to the surrounding draining veins. A scalar opacity algorithm made the bone transparent, and 3D modelling revealed an ED plexus with collecting ducts that drained into the VVA and cranial sinus. From the observed organisation we conclude that this system may constitute the principal outflow system for endolymph in the human ear, and its malfunction may lead to endolymphatic hydrops and MD.

## Material and Methods

### Uppsala temporal bone collection

In total, 113 archival unselected, macerated and 20 fresh-frozen human temporal bones belonging to the Uppsala Temporal Bone Collection were subjected to micro-CT and 3D reconstruction. The ages and the sexes of the donors were unknown. The collection of temporal bones was established in the 1970s and 1980s at the Department of Diagnostic Radiology and Otolaryngology at the Uppsala University Hospital^30,31^.

### Micro-CT

The micro-CT procedures performed on the archival temporal bones has been described in earlier publications^32,33^. Briefly, the bones were scanned with micro-CT (SkyScan 1176; Bruker, Kontich, Belgium) using the following parameters: source voltage 65 kV, current 385 μA, pixel size 9 μm, filter 1 mm Al, exposure time 1 s, frame averaging 2 and rotation step 0.30°. The projection images were acquired over an angular range of 360° with an angular step of 0.3°. The size of the resultant images was 4000 × 2672 pixels and the pixel size was 9 μm. Projections were reconstructed using NRECON software version 1.7.0.4 (Bruker, Kontich, Belgium), based on the Feldkamp algorithm. A volume-rendering technique was used to present a 2D projection of a 3D discretely sampled data set produced by the micro-CT scanner and visualised in a CTvox application (version 3.0; Bruker). Opacity and grey scale values were adjusted to create a realistic 3D view that was as close as possible to that of the real bones.

### SR-PCI

The SR-PCI technique used was recently described by Elfarnawany *et al*.^34^ and Koch *et al*.^35^. The complete ED and ES were not visible in all of the 20 specimens. Five fresh-frozen and then fixed adult cadaveric temporal bones, where the ED and ES were completely visualised, were used in the present study. The 5 bones were from 4 humans (one bilateral). All specimens were obtained with permission from the body bequeathal program at Western University, London, Ontario, Canada, in accordance with the Anatomy Act of Ontario and Western University’s Committee for Cadaveric Use in Research. The bones were thawed and cut to a sample (40 mm diameter, 60 mm length) from each temporal bone. All samples were cut from the middle ear towards the inner ear. The bones were fixed in 3.7% formaldehyde and 1% glutaraldehyde in phosphate buffer for 5 days^36^. The tissue was rinsed and dehydrated in a graded ethanol series. The tissue did not undergo any additional processing (i.e., staining, sectioning or decalcification). Fixative treatment reduced the risk of degradation over the two-month time difference between processing and imaging sessions. Samples were transferred to the imaging facilities in motion-proof containers to prevent damage. The imaging technique used was in-line PCI implemented at the BioMedical Imaging and Therapy (BMIT) 05ID-2 beamline at the Canadian Light Source Inc. in Saskatoon, SK, Canada as described in our precious publications^33,36^. Parameters that are specific to the current image acquisition include: sample-to-detector distance: 2 m, photon energy: 47 keV, detector: AA-60 beam monitor coupled with a C9300-124 camera (Hamamatsu Photonics, Shizuoka, Japan) having 12-bit resolution and an effective pixel size of 9 × 9 µm^2^, imaging field of view: 4,000 × 950 pixels or 36.0 × 8.6 mm, scintillator used in AA-60: P43 (Gd2O2S:Tb) with 10 µm thickness, projections acquired: 3,000 over 180 degrees, exposure time: 100 ms per projection. The final 3D image volume had an isotropic voxel size of 9 µm. The CT data have been reconstructed by NRecon (SkyScan Bruker, Kontich, Belgium). While CT imaging is absorption contrast-based, PCI can potentially be combined with synchrotron imaging to improve soft-tissue contrast while maintaining accurate visualization of bone. Conventional CT based on absorption contrast depends on the attenuation of X-rays, whereas, in PCI, the phase shift caused by the sample is transformed into detectable variations in X-ray intensity. PCI can provide edge enhancement by emphasising the contrast between the boundaries of different structures in the image. The results demonstrate that SR-PCI can be used for simultaneous visualisation of both bone and soft tissue.

### Imaging and analyses

Images were imported into 3D Slicer program (Slicer 4.6; www.slicer.org)^37^, an open software platform for medical image informatics, image processing and 3D visualisation. The surface anatomy of the temporal bone was visualised from the micro-CT images. Images were resized at a scale of 4:1 before 3D reconstruction due to hardware and software limitations. Opacity and grey scale values were adjusted during volume rendering. The technique allows reconstruction of virtual sections and scanned objects in three dimensions. The digitally reconstructed bones could be made transparent and cropped for better visualisation of the VA and its accessory canals. The 3D modelling software was equipped with tools which allowed for geometric measurement in 3D. The dimensions of the VA and VVA were assessed on the five specimens that were visualised with SR-PCI. The lumen of the VA was measured at the internal aperture and at its narrowest point, called the isthmus. Since the internal aperture had an oval shape, two diameters were measured: the shortest and the longest. The lumen of the venous branches of the VVA were also measured. The two main venous branches of the VVA were also measured. The branch that was situated inferior to the ED was designated as branch 1 and the branch superior to the ED was called branch 2. The volume of the VA was determined on five randomly chosen temporal bones visualised with micro-CT. Volume measurement was done by segmentation, which was performed by thresholding determined by visual assessment.

## Results

### Micro-computed tomography (Micro-CT)

**Figure 1:**
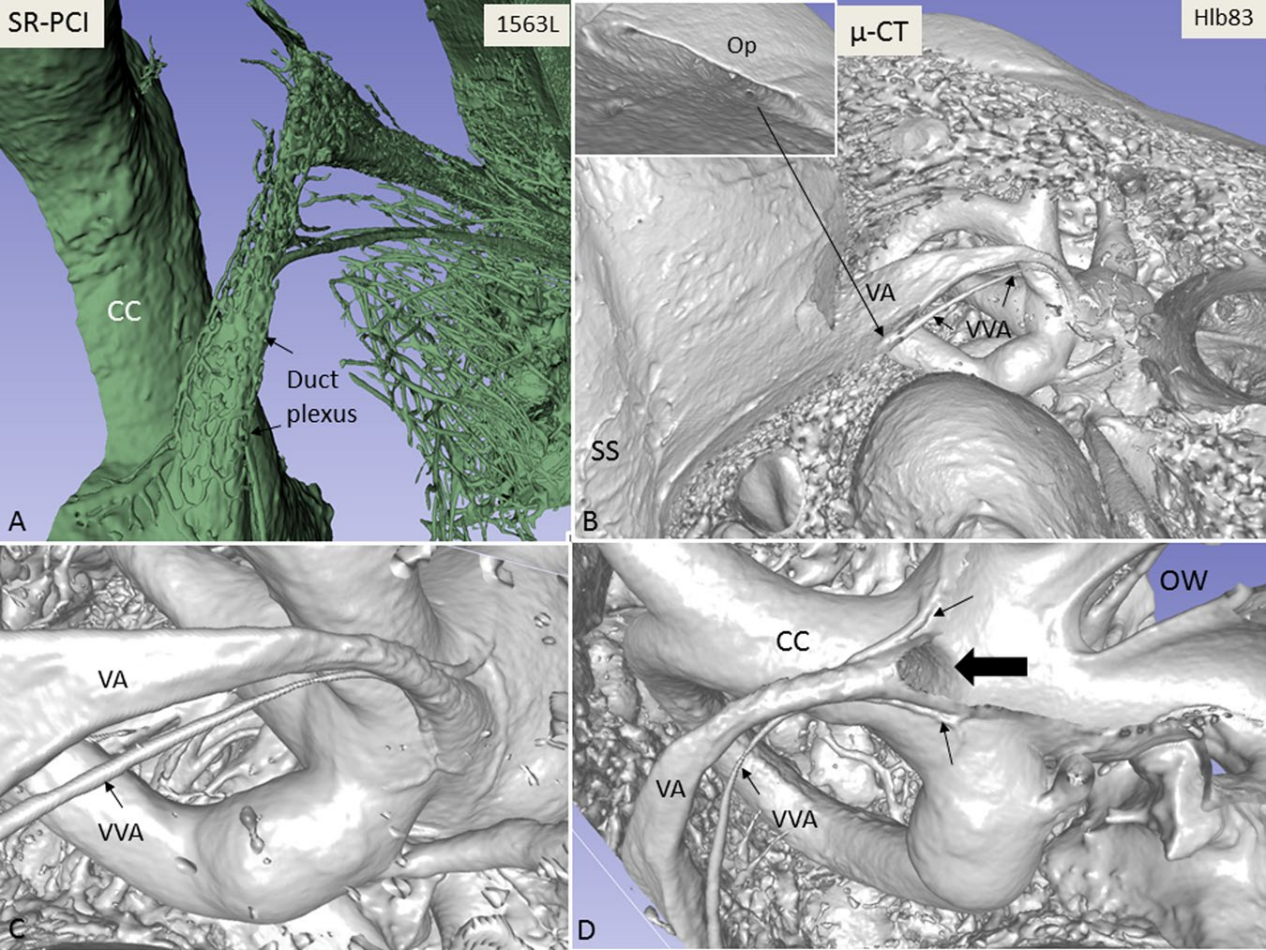
(**A**) Synchrotron radiation phase-contrast imaging (SR-PCI) and 3D reconstruction of a right human temporal bone (anterior view). The structures are visible after the bone was made transparent using a scalar opacity mapping tool. Semi-automatic segmentation visualized the extensive channel system surrounding the VA containing the endolymphatic duct. B-D. Micro-CT of a left human temporal bone. (**B**) shows both the posterior surface of the petrous pyramid and the labyrinth, VA and VVA using a surface algorithm (medial view). Inset shows the external aperture of the VA and the opening of the VVA (posterior view). VVA; vein of the vestibular aqueduct. (**C**) Higher magnification of the VA and VVA from (**B**). No bone vascular plexus can be observed. (**D**) Cropping of VA shows open labyrinth (violet) with internal aperture of the VA (bold arrow) and surrounding veins (small arrows). OW; oval window. CC; common crus. Op; operculum. SS; sigmoid sinus. VA; vestibular aqueduct. VVA; vein of the vestibular aqueduct.

Micro-CT and 3D modelling identified the VA and its accessory canals; however, the soft tissue matter could not be reproduced. Simulated 3D “castings” of the labyrinth were created by a contrast enhancement technique between boundaries. A comparison between SR-PCI and micro-CT reproduction of the VA is shown in Fig. 1. While the bony VA and VVA were well visualised on micro-CT and SR-PCI (Fig. 1B–D), the channel plexus around the ED was only seen in SR-PCI (Fig. 1A). Bone transparency and cropping visualised the 3D bony outline. Extensive variations were noted in the VA and accessory canal anatomy. In general, two to three venous bone channels were observed at the internal aperture of the VA (Fig. 1D). These channels merged into the VVA channel that opened into the floor of the extra-osseous part of the ES (Fig. 1B, inset). The location of the opening of the accessory channel varied greatly but was mostly located at the inferior–medial aspect of the VA. The mean volume of the VA, measured from the internal aperture to the isthmus, was 0.32 µL (range, 0.24–0.43 µL). No difference was detected in the resolution of the tissue between macerated and freshly fixed bones.

### Synchrotron radiation phase-contrast imaging (SR-PCI)

**Figure 2:**
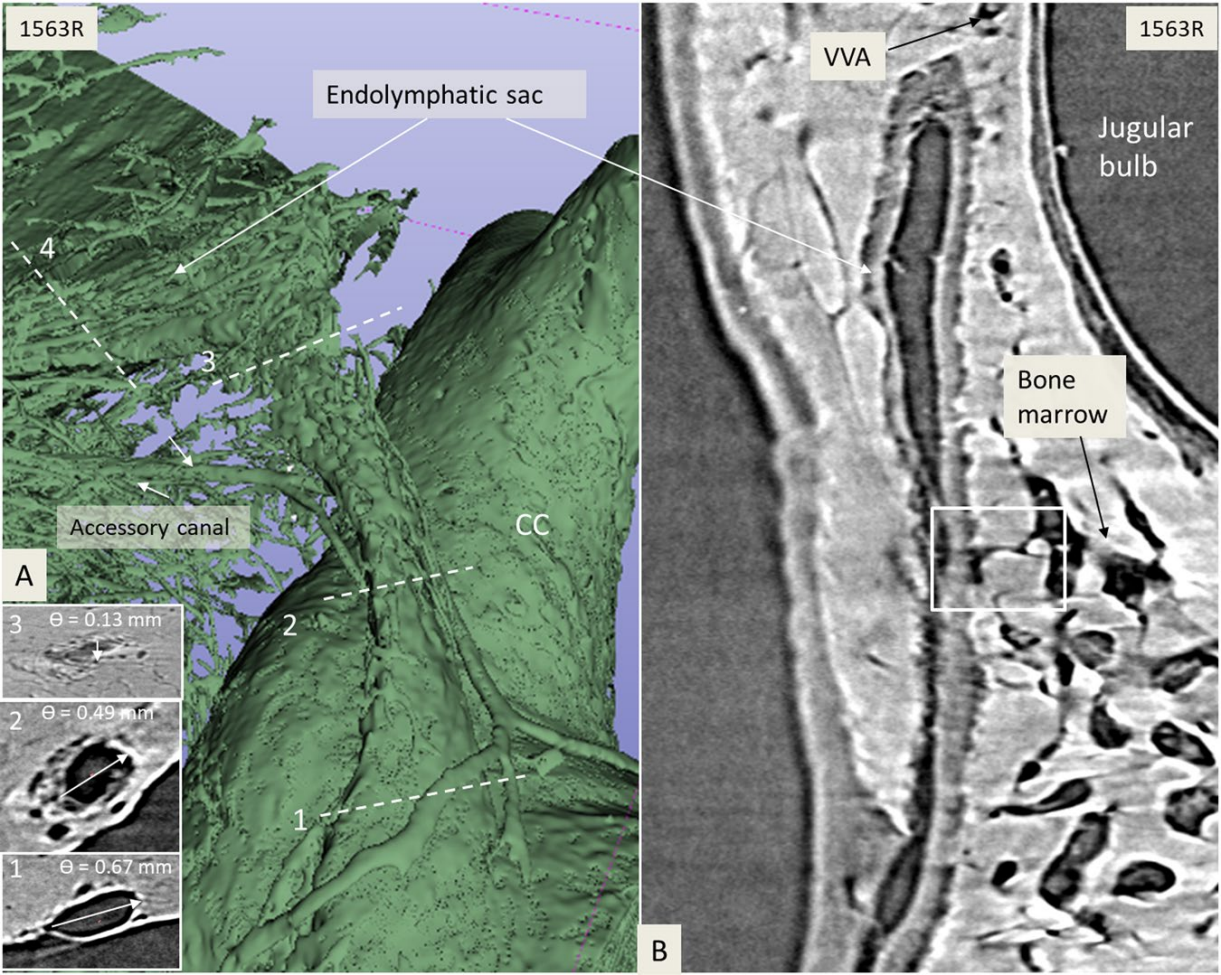
(**A**) Synchrotron radiation phase-contrast imaging (SR-PCI) and 3D reconstruction of a left temporal bone (anterior view). The surrounding bone was made transparent and automatic segmentation shows the channel system surrounding the endolymphatic duct. Transverse sections of the VA are shown in the insets. The accessory canal contains the VVA. (**B**) Section of the endolymphatic sac shows the soft tissue as well as the VVA, which here empty into the jugular bulb (arrow). Framed area shows the connecting channels between the sac and surrounding bone marrow. CC; common crus. VA; vestibular aqueduct. VVA; vein of the vestibular aqueduct.

**Figure 3:**
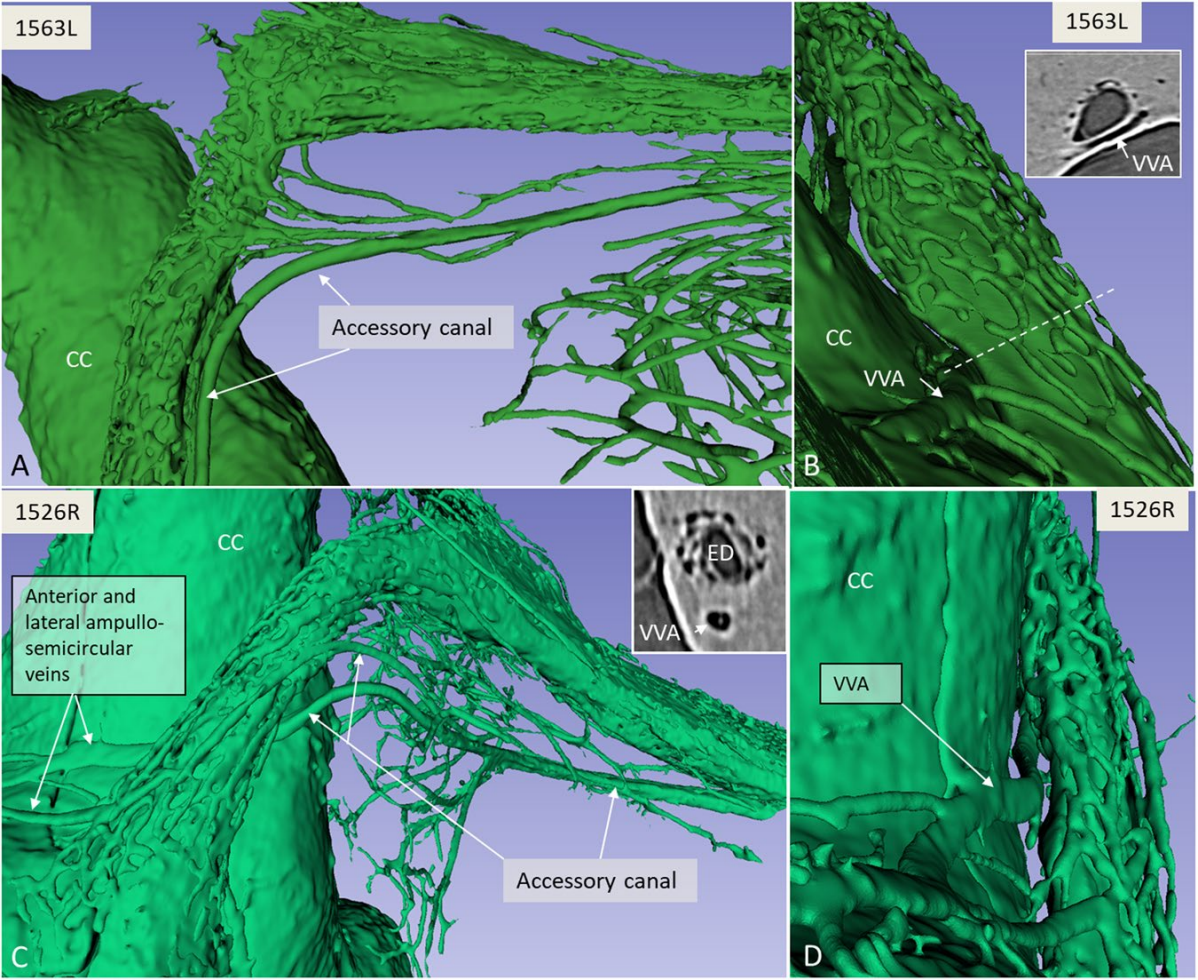
(**A**) SR-PCI and 3D volume rendering of two right temporal bones (**A,B**; 1563L and **C,D**; 1526R, medial views). The surrounding bone was made transparent, showing the channel system segmented around the endolymphatic duct. The plexus drains into the accessory canal at the distal portion of the endolymphatic duct or isthmus. Transverse sections of the VA are shown in insets in B and C. The accessory canal contains the VVA. (**C**) Two veins join into one of the two branches of the VVA. CC; common crus. ED; endolymphatic duct.

SR-PCI and 3D reconstructions of the temporal bones revealed a rich plexus of channels around the VA (Figs. 2 and 3). This was done by making the bone transparent with a scalar opacity mapping tool. This technique enhanced the surface density differences and increased the contrast to visualise small bone channels.

**Figure 4:**
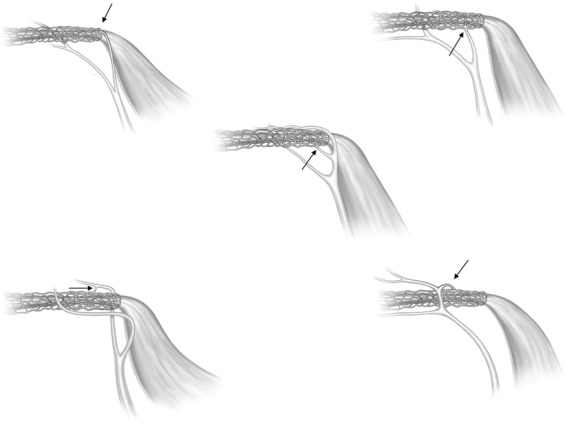
Drawings of the principal organisation of the collecting channels in five human endolymphatic ducts. The site of drainage into the VVA is indicated by arrows.

The ES epithelium and sub-epithelial connective tissue were visualised on X-ray sections. Vessels were sometimes reproduced inside the bone channels. The plexus was located in the bony rim of the VA and was connected to vessels around the ED. Channels were sinusoid-like; they developed proximally and emptied distally into the collecting channels leading to the VVA near the isthmus portion of the VA. The VVA further drained into the sigmoid sinus or jugular bulb. Several channels were typically connected at the distal ED with the accessory canal. Occasionally, the channels were connected to the proximal ES. The VVA was mostly derived from two uniting veins, namely the anterior and posterior branches, as described by Nabeya^38^. The anterior branch was the largest, with twigs from the anterior and lateral ampullo-semicircular and common crus (CC) branches. The posterior branch was derived from the posterior ampullo-semicircular branch and approximated the VA inferiorly. According to Nabeya^38^, the VVA receives branches from all semicircular canals and a part of the vestibule. The VVA anatomy differed depending on the vestibular venous anatomy. Occasionally, the anterior and lateral branches united and ran on the lateral side between the VA and CC. In other specimens the anterior ampullo-semicircular branch ran on the lateral sides of the VA and united with the VVA. The veins sometimes surrounded the VVA on both sides. Various types of anatomy are displayed in Fig. 4.

**Figure 5:**
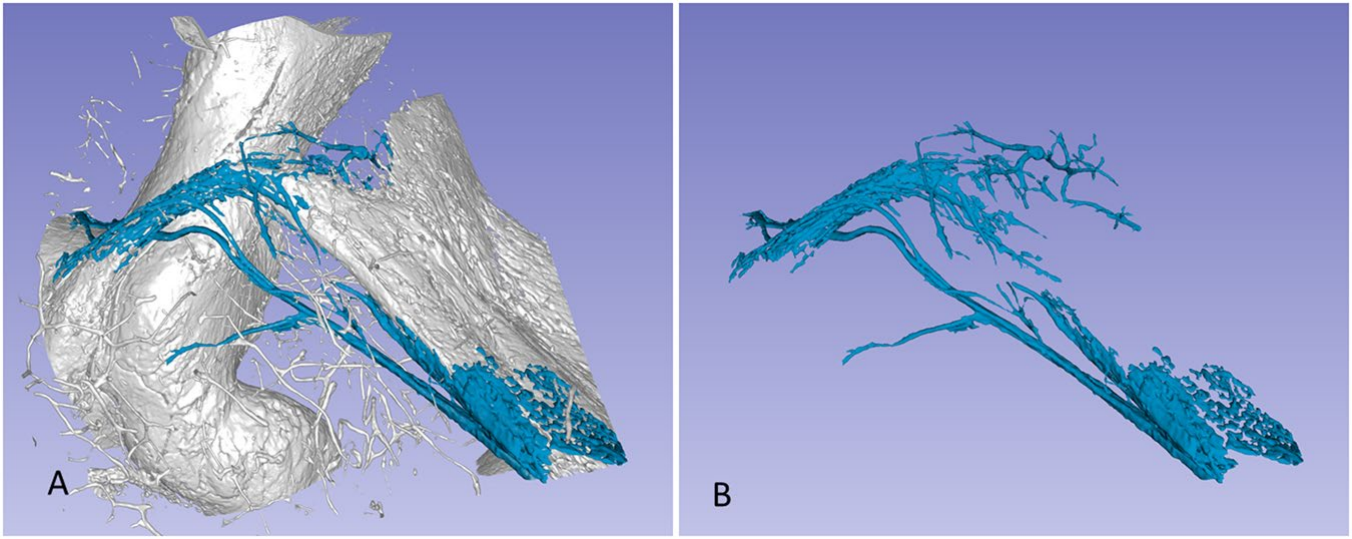
Visualisation of the peri-endolymphatic channels viewed in 3D after segmentation done by manual threshold painting, with (**A**) and without (**B**) the surrounding bone (1526R).

**Figure 6:**
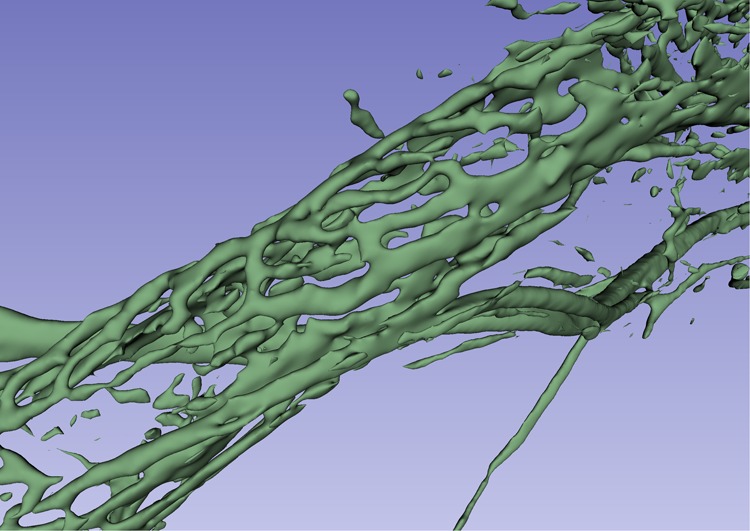
The peri-endolymphatic channel plexus viewed in Fig. 5, but shown in higher magnification. The network consists of a confluent network of sinusoids of different sizes. The VVA passes laterally to the ED. A collecting duct drains into the VVA.

The plexus was not a portal system derived from the VVA but arose locally around the proximal ED and emptied distally into the VVA. In one specimen the channel system was manually modelled (Figs. 5 and 6). Here, branches of the plexus also surrounded the proximal and distal portions of the ES; however, in all cases, the plexus was more developed around the ED. Occasionally channels were interconnected with surrounding bone marrow spaces at the ES.

The ED, at its narrowest point (isthmus), measured 0.18 mm (range, 0.14–0.26 mm), and at the internal aperture, the longest distance was 0.76 mm (range, 0.54–0.88 mm) and the shortest distance was 0.35 mm (range, 0.26–0.54 mm). The diameters of the VVA branches were measured as close to the ED as possible; branch 1 measured 0.08 mm (range, 0.05–0.13 mm) and branch 2 measured 0.07 mm (range, 0.05–0.10 mm) (Table 1).

**Table 1:** Dimensions of the VA and VVA (Measurements in mm).

Specimen	VA Diameter*	VA Diameter** Long	VA Diameter** Short	Diameter of Branch 1 of the VVA	Diameter of Branch 2 of the VVA
2R	0.26	0.81	0.37	0.11	0.10
1526R	0.17	0.75	0.30	0.13	0.05
1563L	0.18	0.54	0.26	0.06	0.07
1563R	0.14	0.83	0.30	0.06	0.09
1571X	0.16	0.88	0.54	0.05	0.05
**Mean**	**0.18**	**0.76**	**0.35**	**0.08**	**0.07**
**SD**	**0.04**	**0.12**	**0.10**	**0.03**	**0.02**

*Diameter of the narrowest part of the VA (isthmus). **Diameter at internal aperture of the VA, shortest and longest distances. The branches 1 and 2 of the VVA were measured at a point nearest the VA. VA; vestibular aqueduct. VVA; vein of the vestibular aqueduct.

## Discussion

In the present study, SR-PCI imaging and volume rendering revealed the 3D organisation of the channel plexus surrounding the human ED for the first time. The plexus is composed of sinusoidal tissue channels, lymphatics and sinusoidal veins. Previously, Ng and Linthicum^39^ and Linthicum *et al*.^29^ generated computer-aided 3D reconstructions from serially sectioned temporal bones embedded in celloidin. They demonstrated an interconnecting system of peri-endolymphatic channels extending from the opening of the VA to the proximal ES and speculated about their hydrodynamic implications. These findings supported earlier observations of a looping system of periaqueductal bone channels, with vessels running in both horizontal and vertical directions and connected to thin periductal vessels^40^. An anastomotic network of thin capillary vessels was observed close to the epithelium of the ED, with vascular tributaries via periaqueductal bony channels. Some, but not all, channels contained vessels. These observations led to the proposal of a unique vascular system of the ED and proximal ES. Non-fenestrated blood vessels were thought to take up fluid and ions, while fenestrated blood vessels surrounding the ES were proposed to mediate macromolecule uptake^41^. Linthicum *et al*.^29^ also presented histopathological evidence of an osseous obstruction of the ED in a case of MD. SR-PCI findings support these results^29,39,40^ and add new information as to the origin and organisation of the plexus containing a distal collecting system. In the present study, collecting ducts were located at the distal ED with the VVA. This organisation also indicates that the plexus plays an important role in draining the endolymph in the human inner ear. An interruption may therefore impede the outflow and cause endolymphatic hydrops and MD. This vascular anatomy resembles the collecting lymph channels that drain the aqueous humour into the aqueous and scleral veins in the eye.

Obliteration of the ES and ED results in endolymphatic hydrops in experimental animal models, indicating that they are essential for endolymph resorption and maintenance of endolymph homeostasis^42^. Kimura *et al*.^43^ even managed to obliterate the reunion duct, with a resulting induction of cochlear hydrops, and saccular collapse with a normal utricle. These, together with the classical experiments by Guild^6^ support the theory of a longitudinal “flow” of endolymph from the cochlea towards the ES via the reunion duct and saccule. According to Salt and DeMott^44^, only large disturbances produce endolymph movements from the cochlea, but they concluded that since local mechanisms in the cochlea are limited, the structures outside the cochlea, such as the ES are important for physiological adjustments of the endolymph volume. Experiments have shown that a change in cochlear endolymph volume results in physiological responses of the ES^15^. The results are consistent with observations of open lymphatics near the epithelium and ED lumen^40,41^. The epithelial tight junctions in the ED are shallow and permeant to ionic lanthanum^28^, while the tight junctions in the ES are more developed, especially in the distal ES^45^. Species differences may limit the value of these experimental studies.

**Figure 7:**
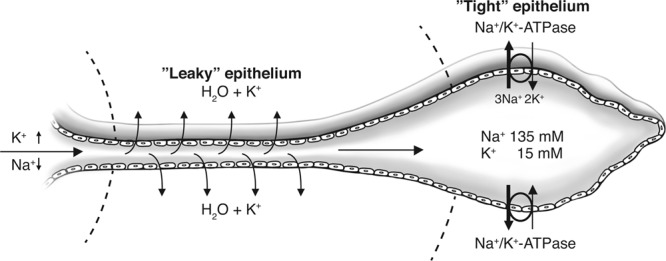
Proposed function of the ED and ES for endolymph absorption. An active Na/K-ATPase pump in the ES epithelium causes a longitudinal “flow” that attracts endolymph in the inner ear. Potassium-rich endolymph reaches the ED and is passively leaked through the ED epithelium into surrounding lymph-venous channels. Due to hydrostatic and osmotic forces, the endolymph reaching the ES is rich in sodium.

Recently, the sodium/potassium-ATPase (Na/K-ATPase) ion transport protein and its isoforms were analysed in the human ES using super resolution immunohistochemistry (SR-SIM)^46^. Antibodies against Na/K-ATPase isoforms labelled a large population of columnar epithelial in the basolateral plasma membrane. Hence, a robust ion pump mechanism is located in the ES and may serve to direct ions and fluid from the rest of the labyrinth to the ES. The endolymph has a unique chemical composition that is suited to the regulation of electrochemical impulses in hair cells. However, wide differences exist in ion concentration, resting potential and pH between the endolymph in the cochlea, vestibule and ES. The resting potential is lower in the ES than in the cochlea but higher in the ES than in the vestibule. Mori *et al*.^47^ reviewed the literature on ion transport regulation in the ES, with clinical implications for MD. In the ES, the potassium (K^+^) and sodium (Na^+^) levels were estimated to 11.6 mM and 103.3 mM, respectively^48–53^. The difference in K^+^ between the cochlea and the ES suggests an outflow somewhere before the endolymph reaches the ES. The absorption of K^+^ in the ED was suggested by Miyamoto and Morgenstern^54^ and others^28,55^. The shallow tight junctions in the ED epithelium may allow leakage of K^+^ and water into the intercellular and sub-epithelial space and into surrounding lymphatics and low-pressure veins^56^. This was demonstrated in Fig. 7.

Aquaporins (AQP) are a family of proteins that form pores in the cell membrane, thus facilitating water transport. Several studies have confirmed the presence of different AQPs in the ED and ES^46,57–59^. AQPs are regulated by vasopressin and studies have shown elevated vasopressin levels during Meniere attacks and reduced endolymphatic hydrops induced by a vasopressin antagonist^60,61^. Interestingly, recent findings show the role of the glymphatic system within the brain that otherwise have been thought to lack a lymphatic drainage^62–64^. New findings show the importance of AQP4 in astrocytes to augment convective flow through the para-vascular space into the interstitial space^65^. Earlier studies from our group showed that the periductal connective tissue cells either express podoplanin, a protein otherwise found on lymph endothelia or a fibroblast marker^66^. An analogous system could thus be represented in the ED by a lymphatic-like channel system closely related to a venous endolymph outflow to the VVA and sigmoid sinus at dura duplicates.

The ES may also act as an active pressure regulator by its secretory capability and could monitor endolymph volume^10–12,67,68^. A hypersecretion in the ES could result in endolymphatic hydrops and MD due to the interference with fluid uptake in the ED. The SR-SIM showed that the β3 isoform, in combination with α1, were expressed with a “reversed polarity” in the apical cell membrane in low epithelial cells^46^. This finding may support the view that the ES can both absorb and secrete fluid.

The etiology of MD has been linked to changes in the ES, such as inflammation, autoimmunity, peri-saccular fibrosis, small size and reduced periaqueductal pneumatization^22,23,69–75^. A reduced absorption of endolymph may be caused by an obstruction or malfunction of the ED. This disruption could be mechanical or instigated by inflammation, leading to periductal fibrosis and density changes in the interstitial ground substance that would in turn reduce the lympho-capillary outflow^56,66^.

Studies on the natural course of MD show an increase in bilaterality with time^76^. It raises query of possible underlying morphologic changes that may predestine for the disease. Such conditions could be hypoplasia of the ED and ES but also abnormalities of the vascular drainage system around the ED. This could lead to EH but also secondary stenosis and obliteration of the ED drainage route.

**Figure 8:**
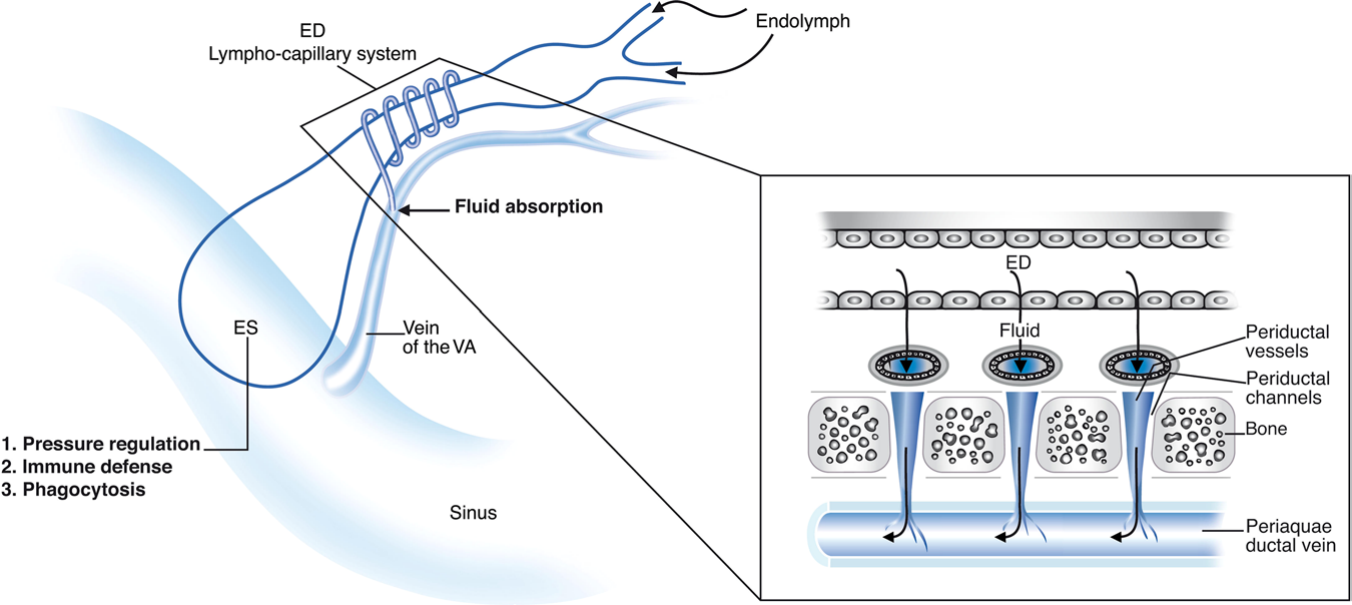
Principal drainage of the vascular system of the human ED and sac. The duct is surrounded by vessels in both the periductal and the periaqueductal space.

Our data suggest that a patent VVA may be a requirement for ED drainage. However experimental obliteration of the VVA in the gerbil did not produce endolymphatic hydrops unless the ES was damaged^13^. In the rat, an obstruction of the VVA distally, just before it empties into the sigmoid sinus, caused a reversed blood flow towards the inner ear^77^; these authors suggested that an obstruction of the VVA could lead to MD. An obstruction or anomalous course of the VVA was described in MD^74^ and a surgical procedure to decompress the ES/VVS to control vertigo and stabilise hearing in MD was presented^78^. The present study provides further evidence that the ED vascular plexus is crucial for endolymph resorption in the human ear and that disturbances may lead to endolymphatic hydrops and MD as presented in Fig. 8. However, more knowledge is needed about the physiological role of the ED and how it can be monitored.

## Conclusion

Synchrotron radiation phase-contrast imaging (SR-PCI) revealed a lympho-venous and sinusoidal plexus around the human ED. The network drained into the VVA and cranial sinus via several collector channels in the distal ED and seemed to form the principal outflow system for endolymph in the human ear. A hypothetical model for human endolymph “flow” and outflow was presented. Its malfunction may result in endolymphatic hydrops and MD.

## Data Availability

The datasets generated or analysed during the current study are available from the corresponding author on reasonable request.
